# CO_2_ and UV Laser-Induced Graphene Based on Polymer Transformation: Advanced Characterizations by 2D Raman Mapping Combined with Microscopy Techniques

**DOI:** 10.3390/ma18133119

**Published:** 2025-07-01

**Authors:** Sabina Botti, Francesca Bonfigli, Alessio Bruttomesso, Federico Micciulla, Valentina Nigro, Alessandro Rufoloni, Angelo Vannozzi

**Affiliations:** 1 ENEA C.R. Frascati, Photonics Micro- and Nano-Structures Laboratory, Physical Technologies and Security Division, Nuclear Department, Via E. Fermi 45, 00044 Frascati, Italy; francesca.bonfigli@enea.it (F.B.); federico.micciulla@enea.it (F.M.); valentina.nigro@enea.it (V.N.); 2 Optoprim s.r.l., Via Dei Quadri 42, 20871 Vimercate, Italy; bruttomesso@optoprim.it; 3 ENEA C.R. Frascati, Superconductivity Laboratory, Fusion Energy Development Division, Nuclear Department, Via E. Fermi 45, 00044 Frascati, Italy; alessandro.rufoloni@enea.it (A.R.); angelo.vannozzi@enea.it (A.V.)

**Keywords:** laser-induced graphene, fabrication process, Raman spectral imaging, confocal laser microscopy

## Abstract

Since its discovery, laser-induced graphene (LIG) has attracted much interest because this technique, having all the advantages of a laser processing technology, is more convenient and cost-effective than other graphene production methods. This work offers a detailed analysis of LIG structures produced by UV and CO_2_ laser irradiation from polyimide performed with surface scanning Raman spectroscopy combined with microscopy techniques. Although UV LIG has a less ordered structure than that obtained by CO_2_ laser irradiation, our study indicates that UV LIG can be patterned with a resolution higher than that obtained with CO_2_ laser irradiation and a much smaller penetration depth into the substrate.

## 1. Introduction

Laser-induced graphene (LIG) is porous graphene obtained by laser scribing a suitable polymer such as polyimide (PI). The laser-induced reactions transform the PI surface into interconnected sheets with a honeycomb structure. Since its discovery, LIG has attracted much interest because this technique has all the advantages of other laser processing technologies, such as controllability, non-contact fabrication, space resolution, and non-catalytic operation [1,2]. Furthermore, LIG can be obtained at room temperature without any polymer pre-treatment, which makes this process more convenient and cost-effective than other graphene production methods. LIG has a wide range of applications due to its unique properties, including high electrical conductivity, large surface area, and biocompatibility.

In 2014 the first LIG was obtained from commercial PI using CO_2_ infrared lasers, circumventing the necessity of high temperatures and vacuum chambers in chemical vapor deposition (CVD) graphene fabrication. Thereafter, several studies have been performed exploring this process with different lasers [3]. Lasers emitting UV wavelengths induce mainly photolysis reactions whereas IR lasers produce pyrolysis processes.

LIG has a three-dimensional structure with pronounced distortion of six-membered rings. Moreover, differently to the honeycomb lattice observed in two-dimensional (2D) graphene, in LIG there is a significant abundance of five- and seven-membered rings [4,5].

The easy production combined with the unique porous structure make LIG a valuable material for future technologies like supercapacitors and batteries with high energy density and fast charging rates, flexible and printable electronic devices, like sensors for activity monitoring, water filtration and other environmental remediation applications [1], and biosensing [6]. This form of graphene is also resistant to biofilm formation [5,7].

In this work, we used both UV and CO_2_ laser scribing to grow LIG from PI. LIG samples were characterized by Raman confocal spectroscopy with surface scanning, confocal laser scanning microscopy (CLSM), scanning electron microscopy (SEM), atomic force microscopy (AFM), and water contact angle (WCA), with the aim to explore the differences between UV and CO_2_ LIG structures. As a precursor, we used PI in the form of Kapton film. Looking at literature data, we found that, although it is possible to obtain LIG from various polymers and organic substrates, PI is the most commonly used precursor [2,3]. In fact, Kapton has a widespread application in aerospace; it is used in spacecraft, satellites, and various space instruments due to its thermal stability, radiation resistance, and low outgassing; and in consumer electronics, it is used in flexible printed circuits, as insulation for wires and cables, and as a substrate for solar cells. Kapton has a significant optical transmittance in the 10.6 µm region and absorbs UV light; therefore, for irradiation with CO_2_ lasers it is better to use thicker Kapton substrate because, due to the photothermal process, a buckling of the substrate is observed [5,8,9]. In this respect, the substrate deformation, leading to non-flat devices, limits the application of LIG [10], while the use of UV light, due the ability to have a more focused spot, allows for both thinner and narrower devices.

The micro-Raman spectroscopy with surface scanning allowed us to study the crystalline structure of graphene along the width of a scribed line or patterned structure, while CLSM provided the surface roughness and penetration depth of laser light for each set of laser parameters. Our experiments indicate that UV LIG has a less ordered structure than CO_2_ LIG, with a smaller pore size in the flakes, although it can be obtained in the form of a laser patterned structure.

## 2. Materials and Methods

### 2.1. LIG Sample Preparation

**Figure 1:**
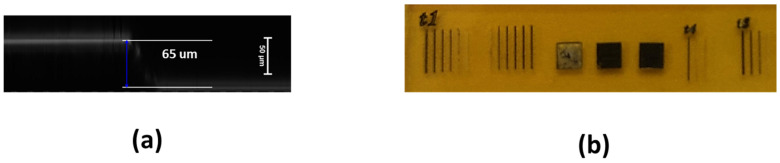
(**a**) CLSM image of xz section of Kapton tape on glass substrate before irradiation. (**b**) Photographic image of LIG structures produced by direct UV laser irradiation of Kapton adhesive orange/brown tape.

As the LIG precursor we used a PI film in the form of Kapton adhesive tape (Dupont, Nemours, France, 16 mm width, about 65 µm thickness) on a glass substrate (see Figure 1). The as-prepared samples were irradiated with a UV nanosecond INNOLAS (Krailling, Germany) laser (maximum power = 5 W, F-theta lens = 100 mm, laser spot ≈ 10 µm, repetition rate 10 Hz) and a CW CO_2_ laser (maximum power = 80 W, F-theta lens = 100 mm, laser spot ≈ 100 µm). Different lines were scribed with varying laser power and scanning speed. LIG surfaces with adjoining lines were obtained, as shown in Figure 1b. The LIG square dimensions of Figure 1b are about (5 × 5) mm^2^.

### 2.2. LIG Characterizations

#### 2.2.1. Surface Scanning Confocal Raman Spectroscopy

Raman spectroscopy is a nondestructive well-established technique for investigating graphene properties. Raman spectra were acquired with a Horiba (Lille, France) XploRA Plus micro-Raman confocal spectrometer operating in the range 100–3500 cm^−1^ with a laser excitation of 532 nm. We used a laser power of 1.5 mW to avoid heat-influenced alteration of Raman spectra and damaging of the samples. To obtain the detailed structural properties, the samples were scanned point by point of a prefixed grid selecting areas down to (5 × 5) µm^2^ using an objective with 100× magnification that gives a laser spot size of about 800 nm and up to (300 × 350) µm^2^ using an objective of with 10× magnification which gives a laser spot size of about 1.7 µm and selecting the “Mosaic” option as the microscope scanning mode.

**Figure 2:**
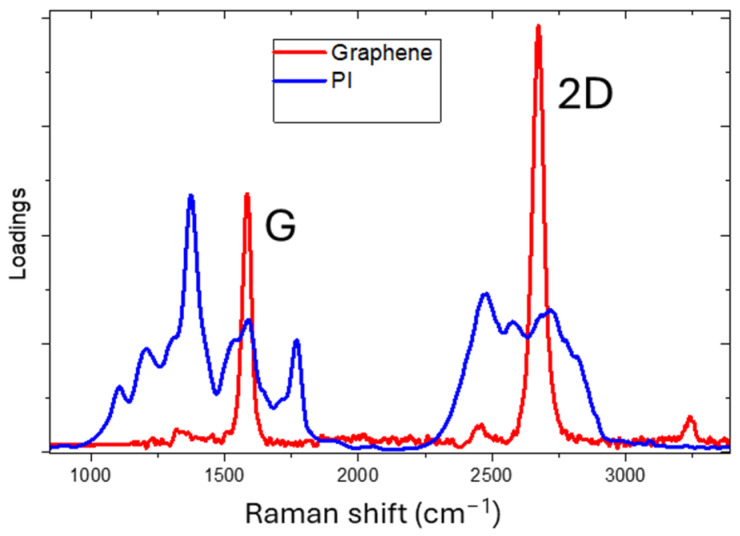
Loading Raman spectra used for CLS fitting: graphene (red curve) and PI (blue curve).

The typical vibration features of graphene are the tangential mode G (1580 cm^−1^) assigned to the stretching of sp^2^ atom pairs, and the D band at 1350 cm^−1^ with its second order (labeled as 2D in Figure 2). While G and 2D bands always satisfy the momentum conservation requirement in the Raman scattering process, the D peak can be observed only in the presence of defects, which provide the missing momentum necessary to fulfill the momentum conservation rule [11,12,13,14]. Therefore, its intensity is proportional to the number of defects in the graphene lattice and, as discussed in the following, the intensity ratio of the D and G bands (I_D_/I_G_) is commonly used for quantifying disorder.

For a given sample the sp^2^ crystalline structure is preserved for a length *L*, which can be considered either as the dimension of in-plane crystallite or as the distance between two neighboring defects [11,12,15,16]. This length can be calculated through the *I_D_*/*I_G_* ratio that is inversely related to the crystallite length *L*, and proportional to the defect density *N*_D_ according to the Tuinstra and Koenig relations:

(1) $$L(nm)=(2.4\cdot 10^{-10})\lambda_{L}^{4}{\left(\frac{I_{D}}{I_{G}}\right)}^{-1},$$

(2) $$N_{D}\left({cm}^{-2}\right)=\left(7.3\pm 2.2\right)\cdot 10^{9}E_{L}^{4}\left(\frac{I_{D}}{I_{G}}\right),$$

where *λ_L_* and *Ε*_L_ are the laser excitation wavelength (532 nm) and the energy (2.3 eV) used for exciting the Raman spectra respectively [11,17,18].

The intensity and shape of the 2D band is sensitive to the z-stacking order of the graphene layers; therefore, the *I*_2*D*_/*I_G_* ratio is widely used to distinguish among single-layer, bi-layer, and multilayer graphene. In the mono-layer graphene, the 2D band is observed to have a symmetric peak, that can be fitted as a single Lorentzian function with a width of 30 cm^−1^ and an *I*_2D_/*I*_G_ intensity ratio larger than 2.0, as reported for non-doped CVD-grown single-layer graphene [11,12,13,14]. The defect density also affects the intensity of 2D band, because defects reduce the electronic lifetime, leading to smaller intensity and larger line width of the Raman peaks [19,20].

The Raman spectra of irradiated samples were fitted through the classical least squares (CLS) fitting method, to calculate the specific contribution of different components, graphene and PI, in the given spectrum. As component (loading) traces, we used the Raman spectra of mono-layer CVD-grown graphene [15] and that of Kapton before irradiation.

Although other components should be considered as residual C-N, amorphous carbon, etc., in a first approximation they can be neglected, and the measured Raman spectra can be properly reconstructed as a linear combination of the two loadings reported in Figure 2. The coefficients of linear combination, named scores, were normalized and used to estimate the percentage of Kapton transformed into graphene.

#### 2.2.2. Confocal Laser Scanning Microscopy

LIG samples were analyzed by a CLSM Nikon (Melville, NY, USA) 80i-C1 operating in laser reflection mode. The samples were illuminated by a 532 nm continuous laser (with a nominal output power of 3 mW). The reflected signal collected by a 20× objective (scanning the sample in a xy field of view of (633 × 633) µm^2^) was detected by a photomultiplier point by point. The CLSM is a significant evolution of the conventional optical microscope. By scanning along the z optical axis, the CLSM performs an optical sectioning of the observed sample by using a pinhole in front of the detector positioned in a conjugate plane with respect to the focus and detecting only signals from the in-focus plane by eliminating signals from out-of-focus ones. The optical sectioning allows obtaining a tridimensional (3D) reconstruction of the sample [21]. By using the optical sectioning operation of the CLSM system, several 2D (xy) slices along the optical z axis with controlled spatial increments (z scanning step = 3 µm) were detected. After acquisition of the 2D slices with a z scanning interval of (0–50) µm and (0–70) µm (depending on the thickness of the samples), 3D reconstructions of the images were performed by the software of the confocal microscope.

#### 2.2.3. Water Contact Angle

The wetting properties of the samples were investigated through a custom-built optical setup for contact angle measurements. The setup includes a fiber illuminator that backlights the sample, which is placed on an adjustable sample holder. A portable Dino-Lite AM4515ZT (Almere, The Netherlands) digital microscope was connected to a computer running the acquisition software (DinoCapture 2.0). This configuration enabled the visualization and the acquisition of magnified droplet images, facilitating contact angle analysis.

#### 2.2.4. Atomic Force Microscope

Topographic surface analyses were performed with a Park System (Suwon, Republic of Korea) XE-Atomic Force Microscope (AFM) operating in non-contact mode. Pre-mounted, non-contact, high-resolution cantilevers working at 309 MHz with a nominal tip radius below 10 nm were used. Images were flattened by subtracting a linear background for the fast scan direction and a quadratic background for the slow scan direction.2.2.5. Scanning Electron Microscope

For SEM characterization we used an Electron Microscope Tescan Vega (Assing, Monterotondo, Italy), with a thermionic source and a tungsten filament. The resolution was 3 nm, with an accelerating voltage of 30 kV.

## 3. Results

### 3.1. UV Laser Irradiation

**Figure 3:**
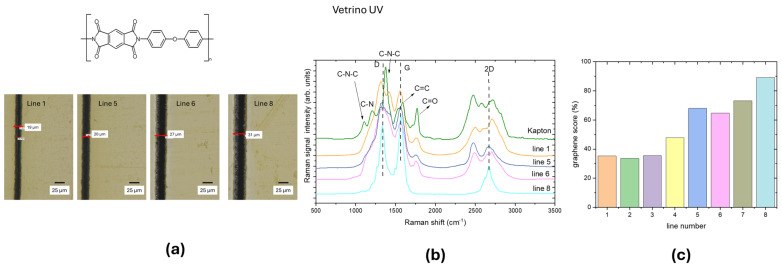
(**a**) Upper panel: polyimide structure (credit: Opera propria, CC BY-SA 3.0, https://commons.wikimedia.org/w/index.php?curid=1552614, accessed on 21 March 2025), lower panel: optical images of lines 1, 5, 6, and 8 written with UV laser at 5 mm/s; the laser power increases from line 1 to 8. (**b**) Raman spectra measured from lines 1, 5, 6, and 8 compared with that of Kapton. (**c**) Graphene scores calculated for the Raman spectra of each line.

To investigate the effect of laser power, LIG lines were scribed keeping the scanning speed constant at 5 mm/s and increasing the UV laser power. The laser power increased from line 1 to line 8. The optical images of selected lines 1, 5, 6, and 8 and the corresponding Raman spectra acquired for each line are reported in Figure 3a,b, respectively.

The Raman spectral features of Kapton PI at 1120 cm^−1^ (C-N-C stretching), 1420 cm^−1^ (C-N stretching) and 1740 cm^−1^ (C=O stretching) progressively disappear from line 1 to line 8 giving rise to D, G, and 2D bands of graphene. These changes indicate that laser irradiation initiates the PI graphitization increasing the C-C bonds and decreasing the C-O and C-N bonds. This is proportional to the laser power.

Following the procedure described in the previous section, the graphene scores were calculated and reported in Figure 3c for each line. From line 5 to line 6 the graphene score increased from 45–65%, pointing to a threshold power that was observed also in UV ablation of polymers [9]. By increasing the laser power, the line width increases, whereas, as observed by other authors, when increasing the scan rate, a higher threshold power is needed to initiate graphitization. Increased laser power leads to higher values of graphene score, up to 90%, proving that laser irradiation of PI material can form the graphene structure breaking nitrogen and oxygen bonds, releasing them as gases.

As already discussed, Raman spectroscopy is a powerful tool to obtain the crystal size (*L*) by analyzing the ratios of the intensities of D and G peaks. For line 8 the *I_D_*/*I_G_* ratio was 0.84 and the L value calculated using Equation (1) was 25 nm, which is comparable to the value of 40 nm obtained by other authors [5]. The obtained L value indicated that the LIG was in the first stage of the amorphization trajectory of carbon materials proposed by Ferrari, in which, due to the presence of defects and disorder in the sp^2^ lattice, graphite progressively transforms into tetrahedral amorphous carbon through two different stages [15,19]. In the first stage the sp^2^ crystalline structure was preserved for a length L and the Tuinstra and Koenig relation held. By further increasing the defect density, the *I_D_*/*I_G_* ratio started to decrease and scaled with L^2^. This unexpected behavior can be explained taking into account that the average distance that an electron−hole pair travels between two Raman scattering events with defects becomes smaller than that between two scattering events with optical phonons; therefore, the contribution of separated defects no longer contributes to the intensity of the D peak. In this second stage of the amorphization trajectory, the nanocrystalline graphene of stage 1 transforms into sp^3^ amorphous carbon. The critical L size that discriminates between the two stages is about 3.5 nm, corresponding to the ratio between the Fermi velocity and the Debye cutoff frequency [19], that is quite a lot smaller than the obtained value of LIG crystallite size.

The *I*_2D_/*I_G_* value was 0.30, indicating that in line 8 multi-layer graphene was formed with a defect density, from Equation (2), of 1.72 × 10^11^ cm^−2^. The defects were mainly due to the high density of edges, derived from the foamy nature of the material. It is worth noticing that in the Raman spectrum there is a large shoulder due to the residual C-N bonds content between the D and G bands. The Raman spectra point to the formation of graphene although we are far from the Raman trace of CVD-grown graphene shown in Figure 2.

**Figure 4:**
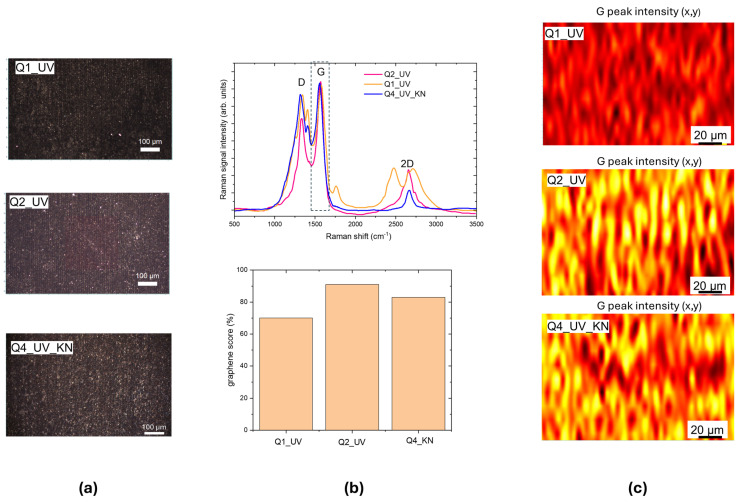
(**a**) White light optical images of UV-written LIG squares: Q1_UV, Q2_UV, and Q4_UV_KN; (**b**) upper panel: Raman spectra measured from Q1_UV, Q2_UV, and Q4_UV_KN squares, the G band is highlighted with a green dot frame, lower panel: graphene scores; (**c**) Raman maps in false color, the contrasting parameter is the intensity of the G peak at 1560 cm^−1^, normalized with respect to the area of whole spectrum. The intensity value increases from brown to red to yellow.

LIG patterned squares with dimensions up to 1 cm^2^ were scribed with different irradiation conditions by using a step size of 10 µm, to obtain adjoined lines. The optical images of UV LIG squares are shown in Figure 4a. The Q1_UV and Q2_UV squares were scribed with the same laser parameters that we used for lines 7 and 8, respectively; for Q4_UV_KN we used the same conditions as for Q2_UV with a faster scanning speed.

The Raman spectra in the upper panel of Figure 4b evidence the formation of graphene with *I_D_*/*I_G_* values ranging from about 0.3–0.45 for Q2_UV. In the latter case an almost total conversion of the Kapton was achieved, with a graphene score of 91%. The Raman maps in Figure 4c are plotted using the intensity of the G peak normalized to the area of whole spectrum as the contrast parameter. In the color code, the intensity increases from brown, through red, to yellow. The yellow stripes correspond to the center of the laser scans.

**Figure 5:**
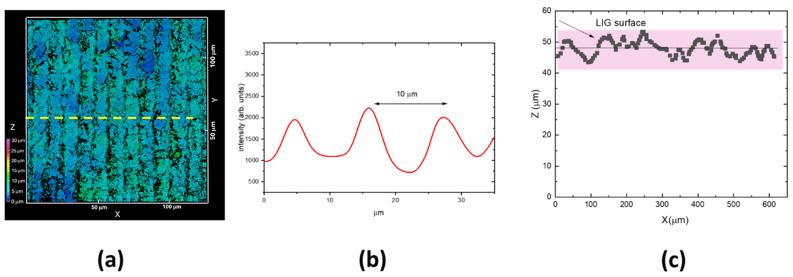
(**a**) 3D CLSM image of the LIG square Q2_UV, xy slice = (130 × 130) µm^2^, z scanning interval 0–30 µm; (**b**) intensity profile along the yellow arrow of (**a**); (**c**) a selected xz slice of (**a**).

Figure 5a reports the 3D CLSM image of LIG square Q2_UV. The LIG structure presented a periodic pattern with a period of about 10 µm, as shown in the graph of Figure 4b, reporting the intensity profile along the yellow arrow of Figure 5a, in correlation with the spot size of the UV writing beam. Figure 5c reports a selected xz 3D projection of Figure 5a. The measured surface roughness is (1.4 ± 0.2) µm, about a fiftieth of the substrate thickness. Therefore, the LIG writing process with focused UV laser beam with a theoretical spot size of about 10 µm is a technique that could be applied to even the thinnest substrates.

**Figure 6:**
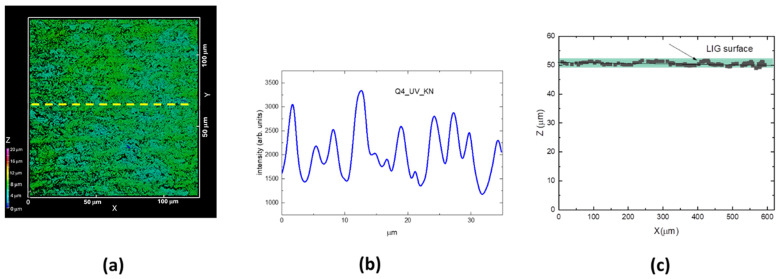
(**a**) 3D CLSM image of LIG square Q4_KN, xy slice = (130 × 130) µm^2^, z scanning interval 0–20 µm; (**b**) intensity profile along the yellow arrow of (**a**); (**c**) a selected xz slice of (**a**).

Due to the faster scanning speed, the stripes in the sample Q4_UV_KN had a smaller width (see Figure 6) and a lower surface roughness: (0.52 ± 0.05) µm.

**Figure 7:**
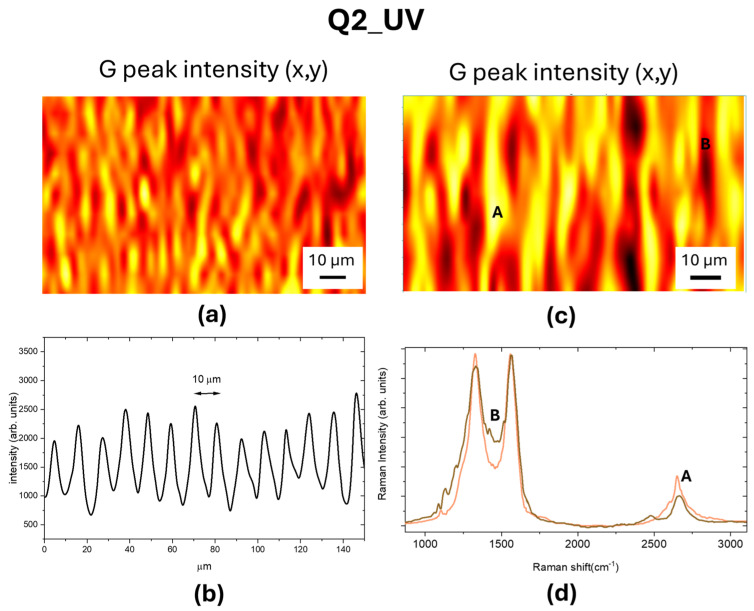
(**a**) Raman map of normalized G peak intensity, the color code is brown-red-yellow; (**b**) intensity profile measured by CLSM of the LIG patterned structures; (**c**) close-up view of Raman map reported in (**a**); (**d**) Raman spectra measured at the point indicated as A of the yellow region and at the point indicated as B of the brown region, in (**c**).

The Raman map of the normalized G peak intensity of LIG square Q2_UV reported in Figure 7a clearly shows that the UV laser wrote graphene with a patterning comparable to that observed by CLSM, reported in Figure 7b. The graphene formation occurred across all the irradiated substrate, as evidenced in Figure 7c,d, with only a slight difference in the *I*_2*D*_/*I_G_* ratio.

**Figure 8:**
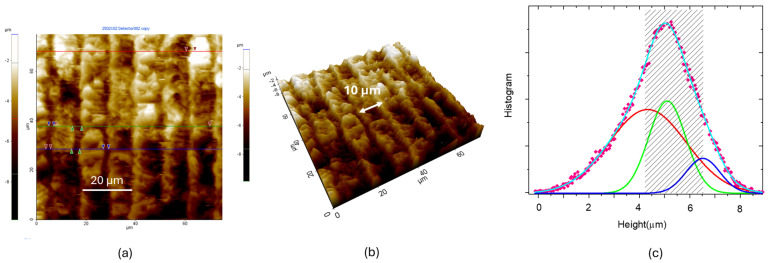
(**a**) Topographic AFM image; (**b**) 3D view of (**a**); (**c**) statistical analysis: height histogram (fuchsia diamonds) acquired from the whole area of the image reported in (**b**). The height distribution can be fitted by three Gaussian functions. The height variation interval is highlighted with a a gray shaded area.

To further inquire into UV LIG samples, we recorded the topography of Q2_UV with AFM, as shown in Figure 8. The LIG patterned stripes had a width of 10 µm and a height ranging from 4.3–6.6 µm, as derived from the height distributions in Figure 8c.

### 3.2. CO_2_ Laser Irradiation

**Figure 9:**
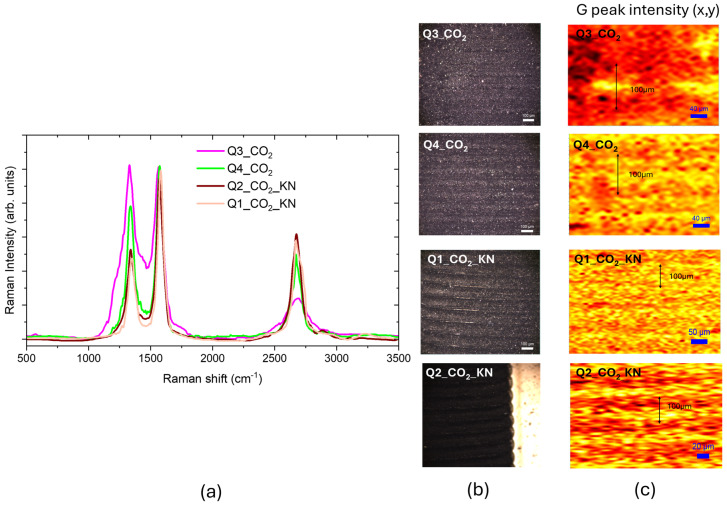
(**a**) Raman spectra of CO_2_ LIG samples; (**b**) white light optical images of CO_2_ LIG samples; (**c**) Raman maps of the normalized G peak intensities of samples imaged in (**b**), the color code is brown-red-yellow.

Figure 9a presents the Raman spectra of the CO_2_ laser-irradiated Kapton surface, imaged in Figure 9b, using different laser powers. By increasing the laser power, the *I_D_*/*I_G_* decreased from 1.0–0.5, improving the crystalline nature of the ablated surface; the shoulder due to the residual C-N bonds decreased, and the increase of the *I*_2*D*_/*I_G_* ratio from 0.3–0.8 suggests a reduction in the number of graphene layers. The crystal size evaluated from (1) was 42 nm, larger than obtained with UV irradiation.

The Raman maps of the normalized G peak intensities, reported in Figure 9c, evidence that the graphene formation was localized only in the laser-scribed regions. In the Q3 and Q4 samples, the scribing laser spot size was 100 µm and the used scanning step was 100 µm. In the Q3 sample, the highest intensity G peak yellow region was in the center of the spot, and when increasing laser power, the G peak intensity was uniform on the spot. For the other two samples, the laser was focused down to 50 µm, obtaining a high G peak intensity across the whole surface; in the sample Q2_KN the adjacent lines were scribed with a step size of 30 µm and we obtained a patterning of the high G peak intensity yellow region that alternated with a lower G peak intensity (orange region). The graphene score calculated with the CLS fitting was 85% for the Q3_CO_2_ sample and about 93% for the others.

**Figure 10:**
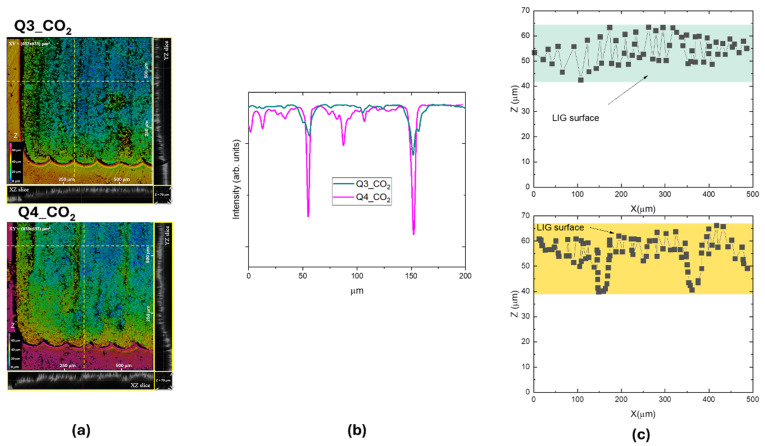
(**a**) 3D CLSM image of a corner of the LIG squares Q3_CO_2_ and Q4_CO_2_; xy slice = (633 × 633) µm^2^, z scanning interval = (0–70) µm. The scale in false colors is from blue (top surface z = 0) to magenta (bottom surface z = 70 µm). (**b**) xy intensity profiles along the dotted horizontal yellow lines of (**a**). (**c**) xz slices with the swelling effect measured in the Z direction.

Figure 10a reports the 3D CLSM images of the Q3_CO_2_ and Q4_CO_2_ samples. In the yellow rectangles of Figure 10a, xz and yz slices selected along the dotted yellow lines are reported showing the z distribution (in a z range of 0–70 µm) of the sample along its thickness. The CO_2_ laser scanning with a 100 µm patterning period is clearly visible in the xy intensity profiles of Figure 10b for Q3_CO_2_ and Q4_CO_2_ LIG patterned squares.

The selected 3D projections are reported in Figure 10c. The surface roughness is (5.6 ± 0.3)µm for the Q3_CO_2_ sample and (6.7 ± 0.2)µm for the Q4_CO_2_ sample scribed at higher power. These are values greater than those of UV LIG, but in any case, about one tenth of the Kapton thickness.

**Figure 11:**
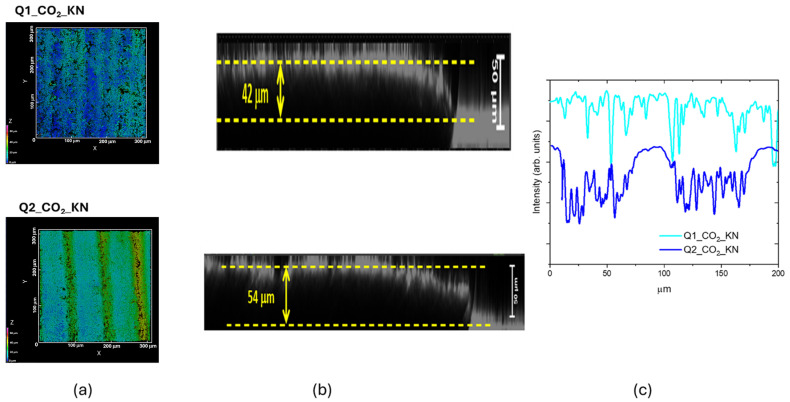
(**a**) 3D CLSM images of Q1_CO_2__KN and Q2_CO_2__KN samples; xy slice = (315 × 315) µm^2^, z scanning interval 0–70 µm; (**b**) xy intensity profiles along horizontal lines of (**a**); (**c**) xz slices with the swelling effect measured in the Z direction.

As shown in Figure 11, we performed the same characterization for the other two LIG samples obtained by CO_2_ laser writing. Figure 11c shows that the treated LIG surface appeared higher (in the Z scanning) than the non-irradiated Kapton surface, showing a rising effect due to the photothermal process. This finding was observed in all the CO_2_ LIG samples. The intensity profile reported in Figure 11b shows a continuous roughened surface for the Q1_CO_2__KN sample, whereas that of Q2_CO_2__KN sample was patterned following the laser scanning step. This patterned structure with an ordered variation in the Z direction could be suitable for growing biological samples. The measured surface roughness values resulted to be (2.7 ± 0.3) µm for Q1_CO_2__KN LIG and (3.8 ± 0.4) µm for the other sample.

### 3.3. Morphological Comparison Between UV and CO_2_ Laser LIG Structures

**Figure 12:**
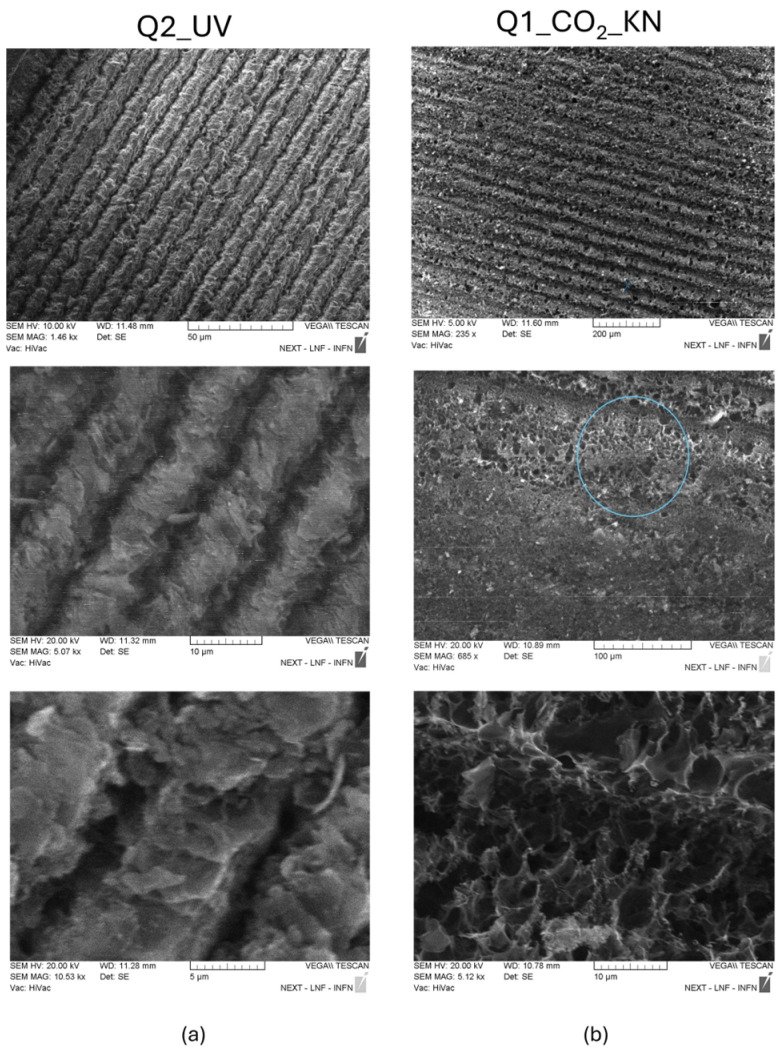
SEM images of UV LIG square Q2_UV (**a**) and CO_2_ LIG square Q1_ CO_2__KN (**b**) at different magnifications. The region highlighted with the blue circle is that magnified in the bottom panel.

The SEM analysis reported in Figure 12 shows the microstructures of the patterned lines in the UV and CO_2_ LIG squares.

In Figure 12a the low-resolution image of the Q2_UV LIG square clearly shows that the stripes of the pattern have a width of 10 µm. The images at higher magnification in Figure 12a report a structure quite different from the foamy structure of CO_2_ laser LIG of Figure 12b: the UV LIG stripes seem to be composed by flat flakes staggered onto each other with pores of few µm. Differently, it can be observed that porous carbon dominates the middle of each pattern of 50 µm width Q1_CO_2__KN stripes. The pores have a larger size that ranges from 5–10 µm.

**Figure 13:**
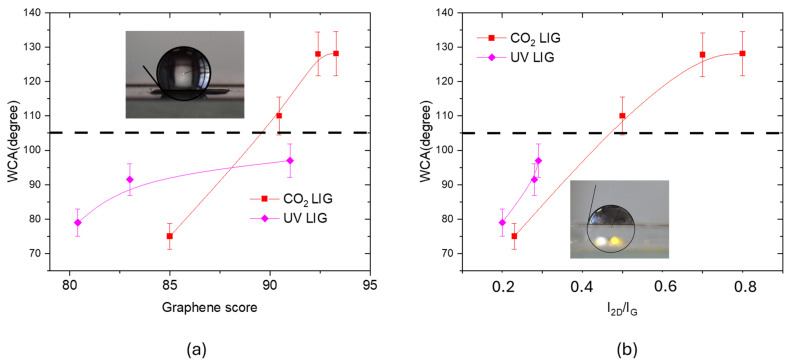
(**a**) WCA measured for UV and CO_2_ LIG samples as a function of the graphene score calculated with CLS fitting; (**b**) WCA measured for UV and CO_2_ LIG samples as a function of the *I*_2D_/*I*_G_ ratio. The dashed lines indicate the WCA value of CVD graphene in both panels.

Such differences in the structures of UV and CO_2_ LIG can explain the variability of measured WCA, shown in Figure 13. As expected, the WCA value increases with the graphene score calculated with CLS fitting and with the I_2D_/I_G_ ratio, namely with the graphene content and with its quality. In fact, graphene has a hydrophobic behavior, with a WCA of (105 ± 5)° [22], which is a complex issue with several contributing factors. The C-C bonds in the hexagonal lattice are nonpolar covalent bonds, the lack of polarity means there are no strong electrostatic interactions with polar water molecules. Moreover, pristine graphene has a relatively low surface energy (around 46–62 mJ/m^2^) [23]. Water, with a high surface tension (around 72 mJ/m^2^ at room temperature), tends to minimize its contact area with low-energy surfaces to reduce its own energy, leading to a high contact angle.

Another aspect to be taken in account for the WCA variability is that the surface energy contribution of oxygen/nitrogen functional groups changes with the percentage of Kapton transformed into graphene.

For similar graphene score values, Figure 13a, the WCA measured for the CO_2_ LIG was about 100° higher than that measured for UV LIG. This finding can be explained by considering that the CO_2_ laser irradiation process creates a porous, three-dimensional network with higher roughness. A rough surface can trap air pockets, minimizing the contact area between water droplets and the solid surface, leading to a high water contact angle and in some cases to superhydrophobic behavior. In fact, the highest value of WCA angle (128°) was measured for the CO_2_ LIG sample with highest roughness (6.7 ± 0.2) µm. Substrates with high WCA are suitable for all the applications that require resistance to biofilm formation.

## 4. Discussion

**Figure 14:**
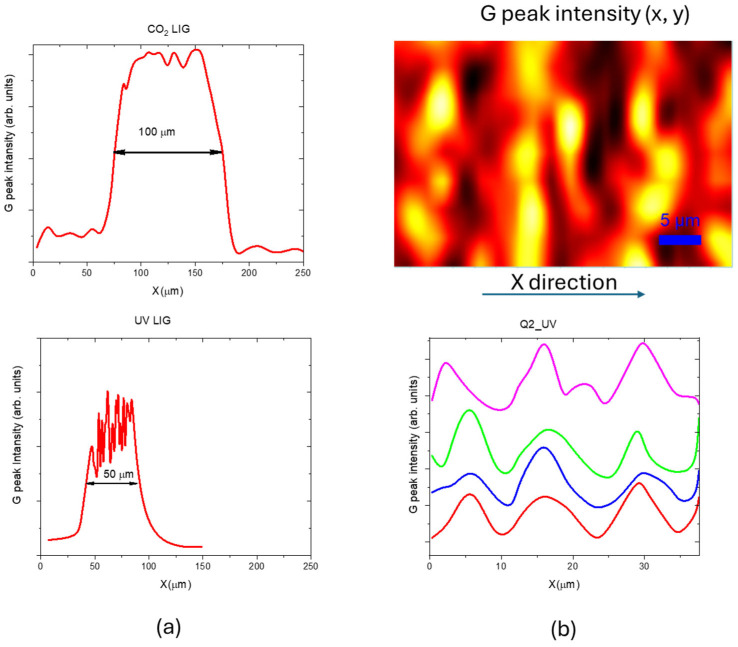
G peak variation along (**a**) the width of lines scribed with CO_2_ and UV lasers using optimized parameters, and (**b**) four lines along the X direction in the Q2_UV samples. The corresponding Raman map was plotted with a step size of 1 µm and 100× objective.

The measured Raman spectra point to the production of lower quality graphene with UV laser irradiation, with a larger content of defects and number of layers. SEM and AFM inspections point to a different structure: the CO_2_ laser facilitated the formation of micrometer-sized pores and a spongy structure, while UV LIG exhibited flakes with limited pore occurrence. The three-dimensional profiles measured by CLSM indicate that a raised structure was formed on the PI by the CO_2_ laser whereas a quasi-planar concave structure was formed on the PI by the UV laser. Such differences can be mainly ascribed to the fact that CO_2_ laser irradiation is a photothermal process with high temperatures that are maintained for longer times, whereas UV irradiation, with its shorter wavelength, enables the photons to possess energy greater than the dissociation energy required for breaking chemical bonds within the material. Notably, the UV LIG had a surface size considerably smaller than that achieved with an infrared laser, as shown in Figure 14, where the variation of G peak intensity along the line width obtained with the two different irradiations is reported. In our experiment, we obtained a patterned UV LIG with a stripe width of 10 µm, as evidenced in Figure 14b. Small conductive traces can be used to fabricate miniaturized LIG sensor arrays for non–invasive wearable sensors [24].

## 5. Conclusions

Confocal Raman spectroscopy and CLSM measurements give a complete characterization of LIG structure, validating the possibility of using a UV laser to induce graphene on the PI surface. Overall, our study demonstrates that, in the explored process parameters range, UV LIG has a less ordered structure that that obtained by CO_2_ laser irradiation. Furthermore, UV LIG can be patterned with a resolution higher than that obtained with CO_2_ laser irradiation and a much smaller penetration depth into the substrate, opening the way for the application of this technology for flexible electronics and non-invasive miniaturized wearable sensors that require planar miniaturized devices.

## Data Availability

The raw data supporting the conclusions of this article will be made available by the authors on request.
